# The implementation and sustainability of a combined lifestyle intervention in primary care: mixed method process evaluation

**DOI:** 10.1186/s12875-015-0254-5

**Published:** 2015-03-17

**Authors:** Brenda AJ Berendsen, Stef PJ Kremers, Hans HCM Savelberg, Nicolaas C Schaper, Marike RC Hendriks

**Affiliations:** Human Movement Science, NUTRIM, School of Nutrition and Translational Research in Metabolism, Maastricht University Medical Centre, PO Box 616, 6200 MD Maastricht, The Netherlands; Health Promotion, NUTRIM, School of Nutrition and Translational Research in Metabolism, Maastricht University Medical Centre, Maastricht, the Netherlands; Internal Medicine, CAPHRI, School for Public Health and Primary Care, Maastricht University Medical Centre, Maastricht, The Netherlands

**Keywords:** Process evaluation, Combined lifestyle intervention, Implementation, Sustainability, Primary care, Overweight

## Abstract

**Background:**

The impact of physical inactivity and unhealthy diet on health is increasingly profound. Lifestyle interventions targeting both behaviors simultaneously might decrease the prevalence of overweight and comorbidities. The Dutch ‘BeweegKuur’ is a combined lifestyle intervention (CLI) in primary care, to improve physical activity and dietary behavior in overweight people. In a cluster randomized controlled trial, the (cost-) effectiveness of an intensively guided program has been compared to a less intensively guided program. This process evaluation aimed to assess protocol adherence and potential differences between clusters. In addition, sustainability (i.e. continuation of the CLI in practice after study termination) was evaluated.

**Methods:**

Existing frameworks were combined to design the process evaluation for our intervention and setting specifically. We assessed reach, fidelity, dose delivered and received, context and implementation strategy. Both qualitative and quantitative data were used for a comprehensive evaluation. Data were collected in semi-structured interviews with health care providers (HCPs, n = 25), drop-out registration by HCPs, regular questionnaires among participants (n = 411) and logbooks kept by researchers during the trial.

**Results:**

Protocol adherence by professionals and participants varied between the programs and clusters. In both programs the number of meetings with all HCPs was lower than planned in the protocol. Participants of the supervised program attended, compared to participants of the start-up program, more meetings with physiotherapists, but fewer with lifestyle advisors and dieticians. The ‘BeweegKuur’ was not sustained, but intervention aspects, networks and experiences were still utilized after finalization of the project. Whether clusters continued to offer a CLI seemed dependent on funding opportunities and collaborations.

**Conclusions:**

Protocol adherence in a CLI was problematic in both HCPs and participants. Mainly the amount of dietary guidance was lower than planned, and decreased with increasing guidance by PT. Thus, feasibility of changing physical activity and dietary habits simultaneously by one intervention in one year was not as high as expected. Also the sustainability of CLI was poor. When a CLI program is started, re-invention should be allowed and maximum effort should be taken to guarantee long term continuation, by planning both implementation and sustainability carefully.

**Trial registration:**

Current Controlled Trials ISRCTN46574304. Registered 23 December 2010.

## Background

Obesity, physical inactivity and unhealthy diet have a combined and independent impact on health [1-4] with increasing social and economic burden. In 2010, overweight related health care costs reached up to 1.6 billion euros in the Netherlands [5]. Accordingly, much effort has been put into promoting healthy lifestyles, resulting in programs ranging from medical treatment to preventive lifestyle interventions.

In general, several studies suggest that combined lifestyle interventions (CLI) aimed at the overweight and obese population yield positive results [6-8]. Unfortunately, such interventions often suffer from high drop-out rates, mainly due to exercise injuries and motivational factors [9,10]. In addition, studies often lack implementation in real world setting [11,12], limiting the generalizability of results to daily practice. Furthermore, sustainability (i.e. continuation in practice after study termination) of lifestyle interventions is crucial to provoke effects on public health. The ‘BeweegKuur’ is a CLI offered by a multidisciplinary team of health care providers (HCPs) in primary care [13,14] and aims at promoting and sustaining both physical activity and healthy diet to improve health of people who have overweight or obesity. In 2007 the ‘BeweegKuur’ has been developed by the Netherlands Institute for Sport and Physical Activity (NISB), commissioned by the Dutch ministry of Health, Welfare and Sports. Over the years, the ‘BeweegKuur’ has been adapted based on process evaluations and now comprises one year guidance by a lifestyle advisor (LSA), physiotherapist (PT) and dietician. The amount of guidance by the PT depends on weight related health risk, based on BMI and presence of comorbidities (see Methods section). A program with six meetings with PT (start-up program) has already been proven effective [15], however, the hypothesized effects of additional guidance (supervised program; 26–34 meetings with PT) remained to be shown. Therefore, the effectiveness and cost-effectiveness of the supervised program compared to the start-up program has been subject of a clustered randomized controlled trial (cRCT) [16]. Thirty primary care health care clusters (HCCs) in the Netherlands participated in the study and were randomly assigned to either the less intensive control program (the start-up program) or the experimental program (the supervised program).

The effectiveness and cost-effectiveness of interventions in primary care depend heavily on process aspects, such as context and delivery of the program. Moreover, process factors may differ between HCPs and HCCs, possibly influencing costs and outcomes [17]. Therefore, process evaluation of complex lifestyle interventions has been advocated, especially in cRCTs [17]. Moreover, studying the process prior to (cost-) effectiveness evaluation ensures a full evaluation of all potential lessons to be learned, instead of a pursuit of explanations for the (cost-) effectiveness outcomes which might introduce interpretation bias [18]. The current study combined parts of several existing theoretical frameworks [17,19-22] to construct a comprehensive structure to evaluate the process of this cRCT of the ‘BeweegKuur’ specifically. By constructing our framework based on existing, generally adopted frameworks, we ensure a full evaluation of the ‘BeweegKuur’ study. In short, our framework consisted of the following concepts: reach and recruitment, fidelity, dose delivered, dose received, context, implementation strategy and sustainability.

The current study evaluated the process of implementation, execution and continuation of the ‘BeweegKuur’ in primary care from both participant and HCP perspective. We aimed to provide insight into possible barriers and facilitators in execution and sustainability of CLIs in primary care, by carrying out the process evaluation prior to the effect and economic evaluation. Furthermore, the process evaluation aimed to gain in depth information for interpretation of the effectiveness and cost-effectiveness evaluation.

## Methods

### Intervention & setting

This study evaluated the process of implementation, execution and sustainability within a multi-center, clustered randomized controlled trial (cRCT) aimed at the effectiveness and cost-effectiveness of two intensities of a combined lifestyle intervention program: the ‘BeweegKuur’ [16]. The ‘BeweegKuur’ is a one-year intervention developed by the Netherlands Institute for Sport and Physical Activity (NISB) and aims at adopting a sustained healthy lifestyle. The ‘BeweegKuur’ consists of programs that differ in intensity of supervision. In this cRCT, the most intensive CLI program has been compared with a less intensive program; the latter has been argued to be both effective and cost-effective [15]. Eligible participants were (1) either overweight or obese (BMI 25–35 kg/m^2^) with at least one of the following serious related comorbidities: sleep apnea, arthritis, cardiovascular disease and/or type 2 diabetes; or (2) morbidly obese (BMI 35–40 kg/m^2^) but without these related serious comorbidities.

Thirty Dutch primary care HCCs were selected by NISB, based on expressed willingness to participate. Each HCC was a collaboration of one or more GPs, LSAs, PTs and dieticians who recruited and/or guided participants. HCCs were assigned at random to the supervised program, or to the less intensive start-up program. HCCs allocated to the start-up program did not offer the supervised program during the current study. Prior to the study, each HCC consented to recruit 20 participants. A detailed description of the intervention and the cRCT is provided in an earlier publication [16]. Both programs comprised six individual meetings with LSA, three individual meetings with a dietician and seven dietary group meetings. In addition, the start-up program consisted of six individual meetings with PT, in comparison, the supervised program consisted of six to seven individual and 26–34 group meetings with PT. It has been hypothesized that the additional amount of guidance within the supervised program increases the effects on physical activity, dietary behavior and health in the population with high weight related health risk. The initial individual meetings with the HCPs were aimed at setting personal goals and identifying barriers to a healthy lifestyle by means of Motivational Interviewing (MI), which were the basis for the further meetings. The PT offered coaching and guidance specifically for physical activity to facilitate transfer to local exercise facilities. At the end of the intervention (12 months after start), the participant had a meeting with LSA to evaluate the lifestyle changes and conclude the intervention.

This study is approved by the Medical Ethics Committee of the Maastricht University Medical Centre and is registered with Current Controlled Trials (ISRCTN46574304).

### Data collection

Process evaluation data were gathered from both HCPs and participants. HCPs of five start-up and five supervised HCCs were selected to participate in face to face, semi-structured interviews. HCCs in both conditions were selected based on relative success of recruitment (low, middle and high recruitment rate), urbanization (rural, municipality and city) and type of HCC (cooperation of geographically separate practices and primary health care under one roof). At the moment of the interviews, the one year intervention was concluded in all participants. Interviews were held with 25 HCPs, of which eight PTs, seven dieticians (of which 2 by phone calls), seven practice nurses with the role of LSA, one dietician with the role of LSA and two PTs with the role of LSA. Two dieticians were not available for the interviews due to personal or organizational reasons. Additionally, every three months, all participants (n = 411) received a questionnaire specifically developed for the current study, which contained items regarding the process. The baseline questionnaire was distributed by the HCP; subsequent questionnaires were distributed and collected via mail by the researchers. In addition, information about drop-outs, reasons for dropping out and loss to follow up were gathered from HCP registries. Moreover, data were extracted from logbooks of informal communication between the HCPs and the research team (registered calls, e-mails and visits to HCC).

### Research framework

Data were collected and presented in a framework which was designed by combining concepts from existing frameworks. Firstly, the RE-AIM framework provided the dimensions reach, efficacy, adoption, implementation and maintenance to illustrate public health impact of an intervention [20]. These dimensions were complemented with key concepts from work by Steckler and Linnan [22] and Saunders and colleagues [21] regarding the evaluation of CLI specifically (fidelity, implementation, dose delivered and received, reach, recruitment and context). In addition, the implementation strategy [19] and clusters were studied to reveal working mechanisms in complex interventions [17]. The specific contents are further elaborated on per concept.

#### Reach and recruitment

Recruitment of clusters (the HCCs) [17] as well as participants were evaluated [21,22]. Interviews with HCPs were aimed at the recruitment procedure (e.g. the HCPs responsible for recruitment and source of participants) and the representativeness of the study population. Participant recruitment was registered per month in all HCCs. HCP measured length and weight, waist circumference and recorded age and sex. HbA1c was assessed and further demographics (nationality, employment, education level and marital status) were retrieved from the participant questionnaires at baseline. Nationality was categorized into Dutch and non-Dutch; employment was categorized into paid work, unpaid work and studying or not working; and marital status was categorized into married, unmarried, cohabiting, divorced and widowed. Education was categorized into low, middle or high based on highest level of completed education. In addition, questionnaires contained items about the recruitment procedure and reasons to participate in the ‘BeweegKuur’ (e.g. ‘What were main reasons for you to participate in the BeweegKuur?’).

#### Fidelity

Fidelity was defined as the execution of the intended characteristics of the intervention [21,22]. The main question addressing fidelity was whether the intervention was implemented consistently with the underlying working mechanisms. MI is one of the main mechanisms of the ‘BeweegKuur’ [13,14,23], and therefore crucial in the fidelity assessment. In addition, setting goals or a plan is essential for lifestyle change. The application of MI and goal setting was discussed in the interviews with the HCPs and also the participant filled in questions regarding goal setting (e.g. ‘Did you set goals with the PT regarding physical activity?’).

#### Dose delivered

Dose delivered described the degree of execution of the program by LSA, PT and the dietician according to protocol [21,22]. The number, content and characteristics of meetings were discussed in the interviews with HCPs. The participants’ questionnaire contained questions about the number of meetings with ‘BeweegKuur’ HCPs every three months (e.g. ‘How often did you have a meeting with the LSA in the past three months?’) and whether planned activities were performed by the HCPs (e.g. ‘Was the BeweegKuur guidance clearly concluded by your LSA?’).

#### Dose received

Dose received was defined as participant satisfaction and perception of the program that was delivered to them [21,22]. Attempted reduction of drop-out and reaction to potential drop-out was discussed in the interviews with the HCPs. In addition, number of drop-outs and reasons were discussed and retrieved from HCP’s own registration, if available. The participant questionnaire contained questions regarding satisfaction with the program and guidance on a scale of 1–10 (10 is best score).

#### Context

Within the context we assessed aspects of the environment with a potential influence on execution and sustainability of the intervention [17,21,22]. Interviews contained discussion about the hindering and promoting factors of continuation of the intervention in the HCC. Also, collaboration to promote participant outflow to exercise facilities were discussed.

#### Implementation strategy

The implementation of an intervention should be planned carefully to facilitate sustainability of change [19]. Implementation was mainly organized by NISB through the Regional Support Structure for Primary Health Care (ROS) [14]. We evaluated the presence of support by ROS and NISB in the implementation and continuation of the intervention in the interviews with HCPs.

### Data analysis

**Figure 1:**
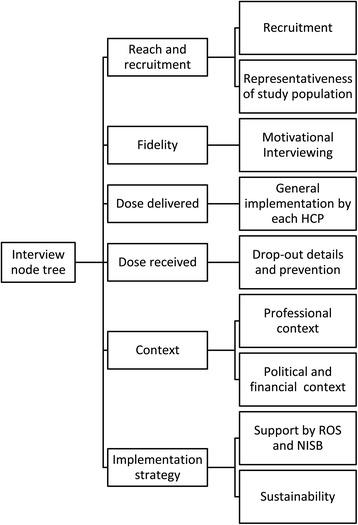
**Node tree with interview contents.**

The interviews were recorded, and a researcher not being the interviewer wrote notes about the content and non-verbal communication. Interviews were transcribed ad verbatim with F4 audio-transcription software (Dr. Dresing & Pehl GmbH, Hamburg, Germany) by a researcher not being the interviewer. Afterwards, transcriptions were read and approved by the interviewer and subsequently made anonymous. Transcriptions were analyzed by means of NVIVO 2.0 (QSR International Pty. Ltd., Warrington, UK) by BB, MH and MS. A node tree was developed based on the study framework to categorize the quotes from the interviews into the specific concepts. The first interview was analyzed with the node tree independently by BB and MH and in case of disagreement between the coding by the two researchers, the node tree was adjusted by deleting, adding or combining nodes. This resulted in a definitive node tree used for the coding of all transcriptions (Figure 1). All codes in transcriptions were read and approved by a different researcher than the coder (BB or MH). BB selected important information from coded transcripts and MH checked the selection of important information from coded transcripts. In case of disagreement, the issue was discussed with SK. Quotes are depicted in the results between quotation marks in italics.

Quantitative data were analyzed in SPSS 21.0 with complete cases for the item of interest (ranging from 135 to 365 participants per analysis). Demographics and questionnaire data were depicted as mean ± standard deviation and in percentages. Differences between the start-up and supervised condition were analyzed with t-tests, Pearson chi square and Mann–Whitney U tests. Differences between HCCs were analyzed with one-way ANOVA or Kruskal-Wallis tests.

## Results

### Reach and recruitment

One start-up HCC dropped out before the start of the study for unknown reasons. One supervised HCC dropped out during the study due to organizational changes in the GP practice; this HCC failed to provide baseline measurements and did not perform any follow up measurement of the participants.

**Figure 2:**
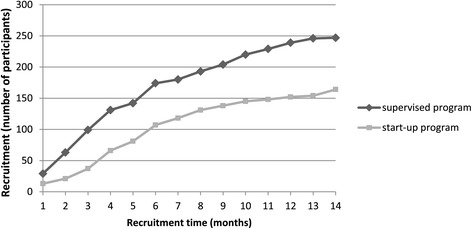
**Cumulative recruitment numbers per month in the two research arms.**

In total, 411 participants were recruited within 14 months, 247 participants in the start-up and 164 in the supervised program, with 2 to 30 subjects per HCC. These numbers were lower than planned and the HCPs declared they had trouble finding suitable subjects, because many potential participants had already been asked to join in the past. In the supervised program recruitment was higher, especially in the first four months (Figure 2). Registries showed that start-up HCCs attributed their low recruitment rate to organizational changes in the HCP team and incorrect information from ROS regarding termination of recruitment. Supervised HCCs with low recruitment gave similar reasons. In addition, start-up HCCs had the possibility to offer the supervised program prior to the study start.

Mean age of participants was 55.1 years (±12.4), the majority was female (64.7%), with Dutch nationality (88.8%) and married (65.6%). Background characteristics did not differ between start-up and supervised participants, except for marital status (p = 0.027; Table 1). Of all participants, 48.9% had diabetes type 2 at baseline, 30.2% did not have diabetes type 2, and of 20.9% presence of diabetes type 2 was unknown (no difference between programs). Mean BMI of the participants was 34.5 ± 4.4 kg/m^2^, waist circumference 113.2 ± 11.2 cm and HbA1c level 6.37 ± 1.12%, with no differences between the two groups.

**Table 1:** **Baseline characteristics of recruited participants**

	**Total**	**Start-up program**	**Supervised program**
	**(n = 411)**	**(n = 164)**	**(n = 247)**
*Sex (%)*			
Male	35.3 (n = 145)	36.0 (n = 59)	34.8 (n = 86)
Female	64.7 (n = 266)	64.0 (n = 105)	65.2 (n = 161)
*Age (mean years ± SD)*	55.1 ± 12.4 (n = 411)	53.8 ± 12.4 (n = 164)	55.9 ± 12.3 (n = 247)
*Nationality (%)*			
Dutch	88.8 (n = 325)	90.9 (n = 130)	87.4 (n = 195)
Other	11.2 (n = 41)	9.1 (n = 13)	12.6 (n = 28)
*Educational level (%)*			
Low	40.3 (n = 146)	37.3 (n = 53)	42.3 (n = 93)
Middle	41.4 (n = 150)	45.1 (n = 64)	39.1 (n = 86)
High	18.2 (n = 66)	17.6 (n = 25)	18.6 (n = 41)
*Occupation (%)*			
Paid work	41.0 (n = 150)	41.3 (n = 59)	40.8 (n = 91)
Unpaid work	22.7 (n = 83)	27.3 (n = 39)	19.7 (n = 44)
Not working/studying	36.3 (n = 133)	31.5 (n = 45)	39.5 (n = 88)
Marital status (%)*			
Married	65.6 (n = 240)	61.5 (n = 88)	68.2 (n = 152)
Unmarried	11.7 (n = 43)	18.2 (n = 26)	7.6 (n = 17)
Cohabiting	9.6 (n = 35)	7.0 (n = 10)	11.2 (n = 25)
Divorced	8.2 (n = 30)	9.1 (n = 13)	7.6 (n = 17)
Widowed	4.9 (n = 18)	4.2 (n = 6)	5.4 (n = 12)
*Body Mass Index*			
Mean ± SD (n)	34.5 ± 4.4 (n = 368)	35.0 ± 4.6 (n = 145)	34.2 ± 4.2 (n = 223)
< 30 kg/m^2^ (%)	16.6 (n = 61)	14.5 (n = 24)	17.9 (n = 40)
30-35 kg/m^2^ (%)	35.6 (n = 131)	33.8 (n = 49)	36.8 (n = 82)
≥ 35 kg/m^2^ (%)	47.8 (n = 176)	51.7 (n = 75)	45.3 (n = 101)

*Significant difference between start-up and supervised participants; p < 0.05.

Baseline data revealed that 48.9% of participants matched the inclusion criteria, 10.0% were healthier (i.e. healthy BMI or no comorbidities) and 16.8% had higher weight related health risk than the targeted population (i.e. BMI of over 40 kg/m^2^ or combination of obesity and comorbidities). For 24.3% of participants eligibility could not be checked, due to missing BMI-value or missing information about presence of comorbidities at baseline. The number of eligible participants did not differ between the programs.

In interviews, HCPs reported that participants were mainly recruited by GP and practice nurse and some HCCs (also) recruited via PT or the dietician. In three HCCs the practice nurse or dietician actively searched through registries to recruit participants; in these HCCs 14, 20 and 21 participants were recruited. In terms of reach per HCP, a practice nurse mainly saw chronic patients, while other HCPs saw more people who had overweight or obesity without comorbidities (‘*I (practice nurse) mainly recruited patients with diabetes, while the GP and PT mainly recruited people who had obesity.*’). If participants were recruited by GP, they often had wrong expectations; this was reported as a possible reason for drop-out by HCPs (‘*Sometimes the GP discussed it too briefly. Well, I think they weren’t very motivated, so I often had to amend participants’ expectations.’*).

According to questionnaires, 76.9% of participants were referred by the GP to LSA for the ‘BeweegKuur’. In total, 80.9% received approval by the GP to start in the ‘BeweegKuur’. The participants reported that their main reasons to participate mainly were to lose weight (n = 242, 58.9%), improve fitness (n = 196, 47.7%), increase physical activity (n = 145, 35.3%), improve health (n = 143, 34.8%), decrease medication use (n = 98, 23.8%) and the combination of both physical activity and diet (n = 87, 21.2%). Only 6.6% (n = 27) reported that improving their current unhealthy eating behavior was a main reason to participate in the ‘BeweegKuur’.

### Fidelity

Except for one PT, all HCPs stated that they were trained in MI techniques and that they applied these techniques in meetings with the participants. HCPs graded their use of MI techniques on average 6.9 (±0.8) on a 10-point scale. There were no differences between type of HCP, HCCs and interventions.

In the interviews, all PTs indicated that they made an exercise plan with the participants (*‘We tried to set up an individual exercise plan based on the Dutch norm for healthy physical activity and several functional tests.’*), while 84.8% of the participants indicated that they set exercise goals or made an exercise plan with an HCP. The majority of the exercise plans or goals were made with PT (79.9%). In total, 90.1% of start-up participants and 93.1% of supervised participants attended at least one meeting with PT, which would be a requirement to set exercise goals. Of six dieticians with whom the topic was discussed during the interviews, five made nutritional plans with the participants. One dietician did not plan individual meetings and therefore felt there was no opportunity to set individual goals. In the questionnaires, 73.9% of the participants mentioned that they made a nutritional plan or set nutritional goals with an HCP. The majority of the nutritional plans or goals were made with the dietician (91.7%). Of start-up participants, 94.4% attended at least one individual dietician meeting essential for setting nutritional goals, in contrast to 63.5% in the supervised program.

HCPs of five HCCs mentioned that the participants often required additional psychological counseling (*‘For a substantial number of participants, the ‘BeweegKuur’ lacked guidance by a psychologist. When it becomes personal, several related emotional matters come up (…), but that was often difficult to expose, because we (as LSAs) have not been trained for that.’*), and according to the HCPs this was due to the shift of target population from patients with type 2 diabetes to people who have overweight or obesity, prior to study start.

HCCs were aware of the study design, and all HCCs were allowed to offer the supervised program prior to the study. Most HCPs from start-up HCCs felt their care had fallen short due to the fact they were not allowed to offer the supervised program to the research population (*‘Although I did not express it to the participants, the fact that certain participants might benefit more from a more intensively guided program did influence my thoughts.’*). In interviews it appeared that HCPs expressed the belief that more exercise guidance was necessary to help this group to adopt a physically active lifestyle. Only one HCP reported that the start-up program had been sufficient for the participants. The planning of individual meetings compared to group meetings and the reduced time investment were seen as advantages of the start-up program by two HCPs.

### Dose delivered

Almost all (96.9%) participants reported that their LSA had explained the intervention clearly at the start of the intervention. Median number of LSA meetings was higher in the start-up program than in the supervised program (Table 2). There was a significant difference in number of LSA meetings between HCCs (range median number per HCC: 0 – 6; Kruskal-Wallis, p = 0.008).

**Table 2:** **Planned and actual dose delivered according to participant questionnaires**

	**Number of meetings according to protocol**		**Attended number of meetings**		
			**(median (25th-75th percentile))**		
	***Start-up***	***Supervised***	***Start-up***	***Supervised***	***P-value***
*LSA meetings*	6	6	4 (2–5)	3 (2–4)	0.017
*PT group meetings*	-	26-34	0 (0–9)	16 (3–24)	<0.001
*PT individual meetings*	6	6-7	2 (1–5)	0 (0–2)	<0.001
*Dietician group meetings*	7	7	2 (0–5)	3 (0–4)	NS
*Dietician individual meetings*	3	3	4 (2–7)	1 (0–3)	<0.001

NS = not significant.

One PT of the start-up program planned group meetings with all HCPs following the intake meeting instead of the intended individual meetings with PT *(‘A one-time advice does not stick. We intensified this by assembling all involved HCPs, to maximize chance of success’*). All HCPs stated that they individualized the program due to either planning issues (holidays), health issues or made well-considered adjustments to individual participants’ wishes and/or needs.

In comparison to the start-up program, the total number of PT meetings was higher in the supervised program (4 and 20 respectively; Mann–Whitney *U* test, p < 0.001), with on average more group meetings and fewer individual meetings (Table 2). The individual PT meetings were not attended by 20.3% of participants of the start-up program and by 53.8% of participants of the supervised program. Within the start-up program, the total number of PT meetings in the intervention period differed significantly between HCCs (range median number per HCC: 0 – 15; Kruskal-Wallis, p = 0.004).

Half of the dieticians had typically offered individual meetings with participants. The other four dieticians planned individual meetings dependent on the participant (*‘For instance, I would say to participants, if you have quite a few questions or you would like some extra support, then I would advise one meeting per month.’*). Main reasons for not planning individual meetings were lack of interest from participants or related costs (*‘Some participants did not want individual dietician meetings, because they had to pay for those meetings their selves.’*). According to the interviews, the number of group meetings ranged from four to eight between dieticians. Two dieticians reported that participants perceived the scheduled seven group meetings to be too much, and therefore planned fewer meetings than prescribed by the protocol (*‘We planned fewer group meetings, just to assure adherence of participants.’*). Four dieticians experienced difficulties in the group dynamics due to background differences between participants, specifically in terms of psychological issues, motivation, age, gender, intelligence and ethnic background (*‘A few participants said the level of the group meetings was too low to attend the meetings.’*). According to the questionnaires, the number of individual meetings in the start-up program was higher (Table 2), but number of group meetings was equal in the two program. The number of participants that were referred to a dietician was significantly lower in the supervised group (82.9% versus 67.5%). There was a significant difference in number of dietician meetings between HCCs (range median number per HCC: 0 – 9.75; Kruskal-Wallis, p < 0.001).

Of 226 participants who completed the questionnaire after 12 months, 40.7% reported the LSA had explicitly concluded the ‘BeweegKuur’ intervention. The intervention was not concluded in 41.2% of the participants and 18.1% did not know.

### Dose received

The participants’ satisfaction with group meetings with PT and with the entire ‘BeweegKuur’ guidance was higher in the supervised group than in the start-up group (Table 3). The satisfaction with guidance by LSA and PT in groups differed between the HCCs (one-way ANOVA, p = 0.018 and p = 0.021).

**Table 3:** **Dose received according to participant questionnaires**

	**Satisfaction (graded 1–10) (mean ± sd)**		
	***Start-up***	***Supervised***	***P-value***
*LSA meetings*	7.2 ± 1.9	7.5 ± 1.7	NS
*PT group meetings*	7.1 ± 2.3	8.0 ± 1.3	0.036
*PT individual meetings*	7.2 ± 2.2	7.7 ± 1.5	NS
*Dietician group meetings*	7.2 ± 2.0	7.1 ± 1.7	NS
*Dietician individual meetings*	7.3 ± 1.9	7.1 ± 1.9	NS
*‘BeweegKuur’ overall*	7.1 ± 1.8	7.7 ± 1.5	0.044

NS = not significant.

Strategies to reduce drop-out consisted mainly of contacting a participant after no show via telephone or mail (reported by 72% of HCPs) and contacting other involved HCPs (28% of HCPs). Two HCPs stated that they deviated from the protocol by adapting the planning of the meetings for individuals with high perceived drop-out risk and three HCPs explicitly discussed the reasons of no show with the participant to prevent future drop-out. Two HCPs were unsure whether they should have put more effort in contacting participants to reduce drop-out, but they had been hindered by time constraints.

According to HCPs, reasons for non-adherence of participants were mainly physical problems or illness (reported by 68% of HCPs), lack of motivation (52% of HCPs), unrealistic expectations towards intervention guidance (*‘Some people might not realize that the ‘BeweegKuur’ requires own effort and activity.’*) or effects (*‘If it didn't quite work for a participant, they could become very critical about the intervention after three or four times.’*; 48% of HCPs), practical issues such as holiday and work (48% of HCPs), group meeting related issues (*‘Some persons did not feel comfortable in the group.’*; 32% of HCPs) and (unexpected) costs of the guidance (20% of HCPs). Less mentioned reasons were low intelligence, private circumstances and that the project was too laborious. Based on the HCP records of each participant and personal communication between participants and researchers, 51 (20.6%) in the supervised program did not complete the planned full year and 38 (23.2%) in the start-up program (based on data of 10 start-up and 15 supervised HCCs and all registrations by researchers). Not all drop-outs of the ‘BeweegKuur’ were registered, but the main reasons were health issues (31.5%) and personal reasons (10.1%).

The interviews revealed that the name of the ‘BeweegKuur’, literally translated ‘Movement Therapy’, could have led to wrong expectations of participants, possibly causing drop-outs *(‘Participants signed up for a movement therapy, so they did not expect nutritional guidance. I think that might have caused drop-outs in the initial phase of the intervention.’*).

### Context and implementation strategy

The ‘BeweegKuur’ was aimed to be covered by the basic health insurance scheme in 2012 [24]. However, this plan was abandoned after a change in government in 2010 [25]. While the initial development of the ‘BeweegKuur’ was ordered by the ministry a few years earlier, the focus of the new minister of Health, Welfare and Sports was less on prevention.

HCPs reported in interviews that support of NISB was mainly experienced in the initial implementation phase of the program, and was perceived to be less present at the time of the execution of study. However, information and material from NISB was continuously used for the guidance of participants. Main functional input from NISB was appreciated in the form of MI and ‘BeweegKuur’ courses, log books for participants, protocols for guidance and participant presents (e.g. water bottles). Support to implement the ‘BeweegKuur’ was mainly provided by the local ROS organizations. Satisfaction of HCPs with this support varied between HCCs. Some HCPs reported that they did not need support by ROS, because the intervention and collaborations were on track or because they did not believe the ROS could provide the help they needed. Other HCPs reported that the ROS did not have a great role in the ‘BeweegKuur’. Support of ROS seemed to reduce after it had become clear that the ‘BeweegKuur’ would not be covered in the Dutch basic health insurance scheme. A few HCPs stated the support was completely terminated and this influenced continuation negatively (‘*All support ceased due to the governmental cutbacks. Then you realize how difficult it is to continue.’*).

The aim of the ‘BeweegKuur’ was that after the one year intervention, participants would continue to exercise in one of the local facilities in the environment of the participant. However, identifying and mapping these facilities by the HCPs was problematic. Indecisiveness and uncertainty regarding whose responsibility it was and time constraints limited the process (*‘We had contact with ROS, because we both held the municipality responsible for the mapping of exercise facilities, but they refused to do that*.’). In some HCCs, the municipality took responsibility to map the exercise facilities, and this was appreciated by the local HCPs. One HCP missed information and material for non-Dutch speaking eligible participants, hindering sufficient guidance for this group.

### Sustainability

None of the HCCs intended to maintain the name ‘BeweegKuur’ specifically after the completion of the study. Four HCCs (40%) still offered a structured intervention to people who have overweight or related comorbidities, based on the ‘BeweegKuur’ (e.g. cardiovascular risk management, type 2 diabetes, chronic obstructive pulmonary disorder). In most of these CLIs, it depended on the participant whether guidance by a dietician was offered. Costs were covered by insurance of physiotherapy and/or dietary counseling, but part of the costs was also frequently paid by the participant. Three HCCs (30%) intended to continue CLIs in their practices and were still in the process of setting this up. Three HCCs (30%) had no intention of continuing a structured CLI; however, in two of these HCCs, the interdisciplinary collaborations were utilized to refer people to PT or the dietician for advice or guidance. HCCs (with the intention of) continuing a CLI, adapted the program to their experiences, their daily practice and the individuals.

HCPs stated that continuation of the ‘BeweegKuur’ or a combined lifestyle program for overweight people was hindered mainly by reimbursement issues (*‘Now that the project is not reimbursed by the government, I have no idea how to finance the ‘BeweegKuur’.’*). A premise for sustainability of CLIs was the availability of funding, such as an affordable participant contribution or reimbursement of program aspects through the health insurance (*‘Reimbursement via the diagnosis-treatment combinations for diabetes still enables us to organize intervention aspects.’*). Another facilitating factor was the collaboration with municipality in the form of local exercise coaches. Most HCPs reported that they were willing to look for funding; however, time constraints hindered them to do so. In addition, a few HCPs had applied for funding of a major health care funding institute; this was either unsuccessful or only postponed the termination of the program.

In most HCCs (60%), the discontinuation of funding led to termination of ‘BeweegKuur’ implementation and execution. Five HCCs explicitly attributed the hindered continuation to changes of the political climate (i.e. less emphasis on prevention). One HCC attributed the discontinuation to organizational changes in their HCC and one HCC was dissatisfied with the CLI in their HCC. Also, the financial situation of participants was seen as hindering by HCPs, as not all participants had sufficient means to cover insurance costs for own account.

## Discussion

The aim of the process evaluation was to provide insight into the implementation, execution and sustainability of a CLI in primary care. A newly composed framework was used to ensure structured and complete evaluation. Both HCPs and participants indicated that the participants’ expectations of the intervention were often not met. Also, guidance was frequently not according to protocol and adherence differed between the two programs and clusters. Nevertheless, in the intensive program people received more PT supervision than in the start-up program. Sustainability of the ‘BeweegKuur’ was low; however, knowledge, experiences and networks from the implementation of the ‘BeweegKuur’ were utilized in most HCCs to continue some form of combined lifestyle approach in primary health care.

Interestingly, a few HCPs stated that information provided by GPs prior to recruitment and the focus on physical activity in the intervention name sometimes led to wrong expectations. In addition, weight loss was the reason to participate in the majority of participants. This is in line with previous findings, showing that participants perceived the intervention to be successful when they lost weight [26]. Weight loss might be a false expectation, because the adoption of physical activity and a healthier diet does not necessarily lead to immediate weight loss [27,28]. Though the goal of the intervention is the adoption of a healthy lifestyle in terms of physical activity and dietary behavior to improve health, not all participants realize this. Therefore, non-adherence in future studies might be reduced if expectations are more realistic and in line with the intervention.

The HCPs rated their application of MI on average a 6.9. An earlier study showed that MI was feasible in primary care and usable in diabetes care management [29]. In addition, MI has been shown to lead to significant weight loss [6]. However, a study evaluating the quality of MI by means of observation, showed that practice nurses applied MI only partially [30], indicating that HCPs may overestimate their skills in optimally applying MI. Regardless, in our study participants were on average very satisfied with guidance by HCPs in the intervention. Participant questionnaires showed that number of PT meetings differed significantly between the start-up and supervised protocol, as anticipated. Although PT guidance should be the only guidance that differs between the two programs, numbers of individual meetings with LSA and dietician were significantly lower in the supervised program. Moreover, the proportion of participants which was referred to the dietician was approximately 15% lower in the supervised program. In addition, interviews revealed that the high amount of PT guidance and the sometimes unexpected nutritional aspects of the intervention might have reduced the number of attended dietary meetings. One could argue that the intensive guidance by PT makes guidance by LSA surplus and dietary change difficult. A study that also concerned a CLI found no effects on objectively measured health behaviors, and authors argued the disadvantage of targeting multiple lifestyle behaviors simultaneously [31]. In a study evaluating implementation in a small amount of ‘BeweegKuur’ HCCs, it had already been observed that guidance by a dietician was not performed according to protocol [32]. Some dieticians indicated that the timing of meetings and their content were possible reasons for non-adherence [32]. The selective rejection of an intervention might have benefits, for instance in terms of feasibility or participant adherence, and is therefore not necessarily undesirable [33]. However, a study by Rutten and colleagues showed that, during the ‘BeweegKuur’, motivation shift for dietary behavior was small, possibly explained by the complexity of dietary behavior. Participants in this study indicated that they were less satisfied with support by the LSA to improve dietary behavior than physical activity [34]. The findings of Rutten and colleagues [34] combined with the low number of attended meetings with the dietician might have caused the lack of motivation after four months of intervention. Even though the numbers of dietician and LSA meetings were lower in the supervised program, our study showed that the participants of the supervised program were more satisfied with the intervention than the participants of the start-up program. Also, some HCPs believed the start-up program did not offer sufficient guidance for all participants. This indicates that it would be preferable to tailor the guidance to individual needs and wishes. However, the difference in effectiveness between the two program intensities and the possible influence of the number of meetings remain to be studied.

The type of participants that was reached seemed to differ between different HCPs who recruited participants. Since recruitment could be performed by all HCPs and this differed between HCCs, the participants’ characteristics might have varied between HCCs, possibly affecting potential costs and outcomes. Nevertheless, relevant baseline characteristics of participants were not different between programs. Cluster randomization reduces risk of contamination and is particularly suitable to evaluate interventions implemented in various locations [35]. A study describing the reach in a cluster randomized trial, showed that recruitment by HCPs who are not blinded, can lead to unequal distribution in the control and experimental group [36]. In our study, motivation between HCCs might have differed, leading to the large variation in number of participants per HCC. This dissimilarity in motivation might have had consequences for program execution during the study and underlines the importance of treating variation between HCCs as potential influence on cost-effectiveness.

Over the years, the ‘BeweegKuur’ has been optimized based on advice from the ministry of Health, Welfare and Sports and on evaluations by Helmink and colleagues [14,26,37,38]. In 2009, the Dutch government intended to include the ‘BeweegKuur’ in the basic health insurance scheme [24]. Process data collected in 2010 showed that HCPs were motivated to implement and continue the ‘BeweegKuur’ [37]. In addition, a study by Rutten et al. [34] showed a shift to a more autonomous motivation for physical activity in ‘BeweegKuur’ participants, which is assumed to precede the engagement in physical activity [23]. However, after a change in government in 2010, the intention of including ‘BeweegKuur’ in the insurance scheme was abandoned [25,38]. According to the interviews in our study, this decision influenced implementation support by ROS, and because they had not anticipated on the lack of funding, the sustainability of this CLI was hindered (i.e. whether participation in a CLI was still possible at the HCC). Our finding that funding and external collaborations were perceived as key factors in sustainability of the CLI in the HCCs, is in line with Green & Tones, who described the impact of lack of funding and collaborations [39]. Although none of the HCCs has actually continued the ‘BeweegKuur’ according to the protocol, most HCCs do offer lifestyle guidance in which strategies, experiences and collaborations from the ‘BeweegKuur’ are employed. Adapting or selectively rejecting parts of an intervention is defined as re-invention, which might support the sustainability of an intervention in daily practice, because the users of the program (i.e. the HCPs) adjust the program to experiences, needs and possibilities of their own and of the participants [33]. The recruitment of participants who strictly would not be eligible for the study, but were recruited anyway, based on HCPs’ experiences, is also a form of re-invention. Nonetheless, most ‘BeweegKuur’ intervention elements are essential for lifestyle change, such as goal setting and evaluation. It is therefore uncertain whether the interventions as they are currently offered will have similar costs and effects as the ‘BeweegKuur’ we have been studying. Also, the low sustainability of the program might be caused by the perceived lack of an implementation strategy [19]. Although most HCPs were satisfied with the support by ROS and NISB during implementation, HCPs missed support in sourcing alternative sources of funding. After the decision not to include ‘BeweegKuur’ in the basic insurance scheme, NISB focused on sustainable networks, and as anticipated, most HCCs still utilized networks formed during ‘BeweegKuur’. During future design and implementation of CLI in real world setting, care should be taken to plan not only implementation, but also sustainability of all aspects of the intervention required for the intended goal.

The HCPs in the current study might not be representative for the entire population in primary care, because they were participating in the ‘BeweegKuur’ from an early stage, and could therefore be labelled as innovators and early adopters [33,37]. Accordingly, sustainability and the extent of program adjustment might be higher due to the longer experience and familiarity with the programs. Another limitation is the implementation of the supervised program in control HCCs prior to this study, which has potentially influenced the degree of re-invention in the control HCCs. For instance, one of the interviewed PTs from the start-up program planned group instead of individual meetings, which might be triggered by the exposure to group meetings of the supervised program prior to the study. However, this reflects the real world setting in which it is unavoidable that previous experiences potentially influence the degree of re-invention of other interventions. In addition, participant registration was used and interviews with HCPs were conducted after the study ended, possibly causing an increased risk of recall bias in both participants and HCPs. Nevertheless, by triangulating information from HCPs and participants in our evaluation, we attempted to minimize effects of recall bias.

The strength of our process evaluation is the application of a solid research framework to identify potential influences on costs and outcomes, but also to provide insights beneficial for future intervention implementation and studies. We have constructed and performed the process evaluation prior to the analyses and interpretation of (cost-) effectiveness, to ensure a full analysis of the factors with potential impact on the results. Also, the triangulation of participant and HCP data increased validity of our results.

## Conclusion

Protocol adherence in our CLI was problematic in both HCPs and participants. Cluster randomization was applied to decrease contamination, but also led to diversity in guidance. Guidance in all HCCs deviated from the protocol, and adherence differed between both programs and clusters. Consequently, we showed that evaluation of (cost-) effectiveness should account for cluster differences, for instance by using multilevel analyses. The high amount of physical activity guidance seems to lead to a diminished opportunity for dietary change, so the guidance in CLIs should be well-balanced to assist multiple behavior change. An important lesson learned is that the liberty of re-inventing the CLI and political and financial facilitation seems to be crucial for the sustainability of the CLI, and should therefore be included in an implementation strategy in future interventions.

## Abbreviations

CLI: Combined lifestyle intervention
cRCT: Clustered randomized controlled trial
GP: General practitioner
HCC: Health care cluster
HCP: Health care professional
LSA: Lifestyle advisor
MI: Motivational interviewing
NISB: Netherlands Institute for sport and physical activity
PT: Physiotherapist
ROS: Regional support structure for primary health care
